# Supplementary material for: Effect of disclosing the relation between effort and unit reliability on system reliability: An economic experiment
Source: PLoS One. 2021 Apr 7;16(4):e0249722. doi: 10.1371/journal.pone.0249722 (PMC8026045; doi:10.1371/journal.pone.0249722)
Supplement: Attachment — Submitted filename: Reply.docx [file pone.0249722.s002.docx]

Dear Dr. Luo-Luo Jiang,

Thank you very much for your judgement. We read very carefully and modify the manuscript following the reviewers’ comments. Additionally, we add the raw data set obtained from the experimental software z-Tree which include all data as the supporting information. Our replies to each reviewer’s comment are below.

Reviewer #1: In this paper, a behavioral experiment is studied, which discloses the relation between effort, rish probability and unit reliability. The results of this experiment are enlightening and worthy of publication.

The only problem is that we hope that the authors can attach the detailed experimental operation process and statistical table to the paper for comparison and reproduction.

Reply) We add the all data in the supplemental material. We show the detailed experimental operation steps by the blue colored sentences in the design and procedure section. We show the subject tables. The statistical results are shown in Table 3.

What's more, in the experiment, are there any new effects and changes in the results if heterogeneous risks are used?

Reply) Line 29-30, 37-38, 210-250, 257-258, and 526-534. Hausken does not consider the heterogeneous risk model. This is very important model when we consider the real world. We cannot discuss the result of heterogeneous model. We add the notification and future issue about this problem by the blue colored sentences in the conclusion section. This is our first priority in the future project.

The figures in the article are very vague, so it is suggested to make them clear.
Reply) We modify the figures.

There are many typos, such as “Error! Reference source not found.” in this paper.

Reply) They are captions of Figures. We modified them.

Reviewer #2: This paper tries to explore the effect of disclosing risk on human decision making, which is definitely an interesting topic. Based on the integrated models of PRA and game theory, which is initiated by Hausken(2002), the authors developed their experiments to find insights. However, what is the contribution of this paper on Hausken(2002) is unclear. In other words, the motivation of this work should be highlighted.

Reply)　Line 39-47, 51-52, 54-58, 169-170, and 189-200. We clarify the motivation and contribution of this study in red colored sentences in the introduction section. Hausken only consider the theoretical equilibrium in one-shot game under the complete information condition. Our study conducts laboratory experiments to evaluate whether the game achieves the equilibrium by actual human subjects. We also extend this model into repeated games to test find what happens after they experience the accident and know the counterpart’s effort as shown in Tables 4 and 5.
